# Cognitive and Brain Activity Changes After Mnemonic Strategy Training in Amnestic Mild Cognitive Impairment: Evidence From a Randomized Controlled Trial

**DOI:** 10.3389/fnagi.2018.00342

**Published:** 2018-11-13

**Authors:** Sharon S. Simon, Benjamin M. Hampstead, Mariana P. Nucci, Fábio L. S. Duran, Luciana M. Fonseca, Maria da Graça M. Martin, Renata Ávila, Fábio H. G. Porto, Sônia M. D. Brucki, Camila B. Martins, Lyssandra S. Tascone, Edson Amaro, Geraldo F. Busatto, Cássio M. C. Bottino

**Affiliations:** ^1^Old Age Research Group (PROTER), Department and Institute of Psychiatry, Faculty of Medicine, University of São Paulo, São Paulo, Brazil; ^2^Division of Neuropsychology, Department of Psychiatry, University of Michigan, Ann Arbor, MI, United States; ^3^VA Ann Arbor Healthcare System, Ann Arbor, MI, United States; ^4^Neuroimagem Funcional – Laboratory of Medical Investigations on Magnetic Resonance Imaging (LIM-44), Hospital das Clinicas HCFMUSP, Faculdade de Medicina, Universidade de São Paulo, São Paulo, Brazil; ^5^Laboratory of Psychiatric Neuroimaging (LIM-21), Department and Institute of Psychiatry, Faculty of Medicine, University of São Paulo, São Paulo, Brazil; ^6^Department of Neurology, Faculty of Medicine, University of São Paulo, São Paulo, Brazil; ^7^Department of Preventive Medicine, Paulista School of Medicine, Federal University of São Paulo, São Paulo, Brazil

**Keywords:** memory training, cognitive rehabilitation, mild cognitive impairment, Alzheimer’s disease, neuroimaging, functional MRI

## Abstract

**Background:** Mnemonic strategy training (MST) has been shown to improve cognitive performance in amnestic mild cognitive impairment (a-MCI), however, several questions remain unresolved. The goal of the present study was to replicate earlier pilot study findings using a randomized controlled design and to evaluate transfer effects and changes in brain activation.

**Methods:** Thirty patients with a-MCI were randomized into MST or education program. At baseline, participants completed clinical and neuropsychological assessments as well as structural and functional magnetic resonance imaging (fMRI). Interventions were administered individually and comprised four sessions, over 2 weeks. MST taught patients to use a three-step process to learn and recall face-name associations. Post-treatment assessment included fMRI, a separate face-name association task, neuropsychological tests, and measures of metamemory. Behavioral (i.e., non-fMRI) measures were repeated after one and 3-months.

**Results:** Participants in the MST condition showed greater improvement on measures of face-name memory, and increased associative strategy use; effects that were accompanied by increased fMRI activation in the left anterior temporal lobe. While all participants reported greater contentment with their everyday memory following intervention, only the MST group reported significant improvements in their memory abilities. There was no clear indication of far-transfer effects to other neuropsychological tests.

**Conclusion:** Results demonstrate that patients with a-MCI not only show stimulus specific benefits of MST, but that they appear capable of transferring training to at least some other cognitive tasks. MST also facilitated the use of brain regions that are involved in face processing, episodic and semantic memory, and social cognition, which are consonant with the cognitive processes engaged by training.

## Introduction

The world population is aging, which leads to an increase of age-associated conditions such as Alzheimer’s disease (AD) and related dementias (Jorm and Jolley, 1998; Prince et al., 2013). Mild cognitive impairment (MCI) is considered the transitional stage between normal aging and dementia, characterized by cognitive impairment in the absence of impaired daily functioning (Petersen et al., 2001, 2014). The clinical presentation of MCI is classified as amnestic or non-amnestic (Winblad et al., 2004), and those patients with amnestic MCI (a-MCI) are at increased risk of conversion to dementia (Espinosa et al., 2013), especially due to AD (Petersen et al., 2001; Jungwirth et al., 2012). Given the predicted multifold increase in the prevalence rates of dementia worldwide, early identification and treatment of individuals at risk of progressing to dementia is imperative.

There is a growing interest in non-pharmacologic treatments for MCI due to the limited benefits of existing pharmacologic agents (McGhee et al., 2016), and the recognition that cognitive interventions can promote brain plasticity and reduce cognitive impairment (Belleville et al., 2011). Also, older adults who are more engaged in cognitively stimulating activities present reduced risk of cognitive deterioration and dementia (Verghese et al., 2003; Barnes and Yaffe, 2011; Marioni et al., 2012), indicating that is it critical to develop effective cognitive interventions for this population.

Decline in episodic memory is one of the hallmark features of AD (Sperling et al., 2010) and is present in a-MCI, which also show deficits in associative memory (Oedekoven et al., 2015) and strategy use (Ramakers et al., 2010). Mnemonic strategies can be broadly conceptualized as cognitive methods that facilitate the organization and association of new information, thereby enhancing depth of processing (Hampstead et al., 2014). Previous literature suggests that mnemonic strategy training (MST) can be effective for patients with MCI, leading to improvements in different cognitive domains (for reviews Simon et al., 2012; Huckans et al., 2013; Hampstead et al., 2014; Chandler et al., 2016; Mewborn et al., 2017). It is worth mentioning that training gains in older adults are more likely to transfer to similar measures in the same trained cognitive domain or construct (“near-transfer effect”), than to operations in non-trained cognitive abilities or domains (“far-transfer effect”) (Mewborn et al., 2017).

Besides transfer effects to objective cognitive measures, training studies have investigated the effects on subjective aspects of memory, such as metamemory, which includes knowledge, perception, and beliefs about one’s own memory and memory functioning (Flavell and Wellman, 1977; Hertzog et al., 1990). Some studies have reported metamemory gains after cognitive training in MCI patients, including greater satisfaction with everyday memory (Bahar-Fuchs et al., 2017), greater self-report memory ability (Rapp et al., 2002; Belleville et al., 2006), and greater self-report strategy use (Rapp et al., 2002; Troyer et al., 2008; Kinsella et al., 2009; Jean et al., 2010; Bahar-Fuchs et al., 2017). The relation between cognitive training-related gains and metamemory is still unclear and deserves further investigation. We hypothesize that memory training would lead to a more positive feelings about memory abilities, and more knowledge about memory functioning.

Several neuroimaging studies have revealed that MST (re)engages brain regions associated with executive functions and memory, with evidence of increased task-related activity in brain areas not recruited before training in those with MCI (Belleville et al., 2011; Hampstead et al., 2011; Belleville and Bherer, 2012; Hosseini et al., 2014; Balardin et al., 2015). For instance, Belleville et al. (2011) found training-related increase of activation in the right inferior parietal lobule after training several memory techniques. Balardin et al. (2015) found the increase of activation in the ventromedial prefrontal and right superior frontal gyrus after teaching a specific semantic strategy. Regarding MST focused on face-name association, Hampstead et al. (2011) reported an increase of activation in a widespread cerebral network involving medial frontal, parietal, and occipital regions, and the left lateral temporal cortex. Likewise, Hampstead et al. (2012b) demonstrated a partially restorative effect of MST on hippocampal activation in those with MCI. Altogether, these findings provide further evidence of neuroplasticity in those with MCI and suggest that MST enhances memory through a mixture of restorative and compensatory mechanisms.

Everyday memory deficits in MCI typically involves difficulties in recall recent information, such as appointments, duties, localization of personal belongings and names of people (Irish et al., 2011; Yanhong et al., 2013). A promising avenue of research is the MST focused on face-name association, since forgetting names is common and has direct social implications (James et al., 2008; Weaver Cargin et al., 2008). Also, memorizing faces has been considered cognitively challenging due to the uniqueness and arbitrariness of the faces (Werheid and Clare, 2007). Neuroimaging findings suggest that face-name binding implicates associative occipito-temporal cerebral regions with extensive connections to other cortical and subcortical brain regions critical to episodic memory (Sperling et al., 2003; Petrella et al., 2006; Chua et al., 2007). These same regions are known to be affected in MCI (Whitwell et al., 2007). Face-name associative tasks have demonstrated sensitivity to memory impairment related to MCI-AD spectrum (Clare et al., 2002; Parra et al., 2010; Polcher et al., 2017), and deficits in such tasks have been associated to Aβ burden in clinically healthy older adults (Rentz et al., 2011). Thus, training those with MCI to enhance memory for faces and names may yield positive everyday effects (i.e., ecological relevance). While face-name associations have been targeted by many studies, most have used a mixture of techniques (Clare et al., 2009; Jean et al., 2010; Belleville et al., 2011) or are uncontrolled studies (Hampstead et al., 2008), which limit knowledge about whether MST is effective. In addition, although there is some evidence of increase of brain activation in a widespread cerebral network after face-name association training (Hampstead et al., 2011), additional research is necessary to better understand the neural mechanisms underlying intervention effects. Moreover, the existing studies have been limited to North American or Northern European samples but there may be important cultural differences that render MST less appropriate.

In the present study, we detail the cognitive and neuroimaging results of a randomized, controlled, single-blind study in which patients with a-MCI were assigned to either MST (focused on face-name association) or an active control intervention (education program – EP). Our primary aim was to examine the efficacy and persistence of MST for face-name associations in a Brazilian cohort, by examining near-transfer effects to face-name tasks. Secondary aims included: (1) to investigate the neurobiological mechanisms associated with MST near-transfer effects using fMRI; and (2) to investigate far-transfer effects on neuropsychological tests and metamemory. Based on the literature, we hypothesized near-transfer effects that would be accompanied by increase of brain activation and metamemory gains, whereas cognitive far-transfer effects were considered less likely. To enhance near-transfer, our MST program incorporated a novel and ecologically vital step where patients applied the methods using real individuals from their daily lives.

## Materials and Methods

### Experimental Design and Sample Size Calculation

**FIGURE 1:**
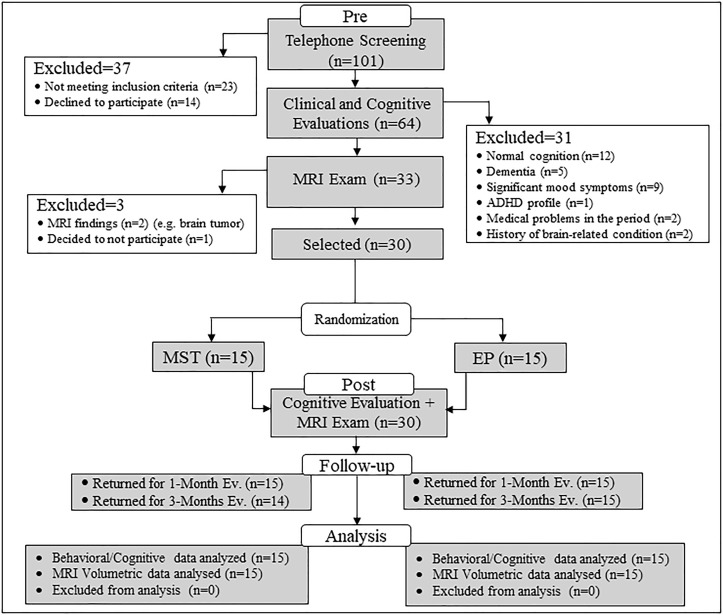
CONSORT flowchart for selection of study participants. ADHD, attention deficit hyperactivity disorder; CSF, cerebrospinal fluid; ED, education program; MRI, magnetic resonance imaging; MST, mnemonic strategy training.

Subject flow is shown in Figure 1 according to the CONSORT diagram (Schulz et al., 2010). Participants were randomly assigned by an independent researcher to one of the two intervention programs (MST or EP). The study protocol was approved by the Ethics Committee of the Medical School of University of São Paulo and registered in ClinicalTrials.gov (NCT01978353). All participants were volunteers and provided written informed consent in accordance with the Declaration of Helsinki. During the consent process, subjects were informed that the study examined the efficacy of various programs for memory; however, they were given information about their specific treatment only after randomization (i.e., participants remained blinded to the other condition). To assess immediate training effects, participants were evaluated approximately 1-week after finishing the programs (average 5.6 days), and maintenance of the effects was evaluated in follow-up assessments at 1-month and 3-months.

The sample size was calculated *a priori* with G-Power software V. 3.1 (Faul et al., 2007). We found a large effect size ($\eta_{p}^{2}$ = 0.89) in our previous uncontrolled pilot study of MST for face-name associations (Hampstead et al., 2008) and used it to power the current study, focusing on the immediate post-intervention timepoint since our control condition (i.e., EP) lacked the “active” ingredient of MST. The resulting two-group analysis revealed a sample of eight participants was needed to detect an effect with 0.95 power at an alpha level of 0.05. Since this seemed rather optimistic, we then conducted a sensitivity analysis that demonstrated a total sample of 30 would be sensitive to detect a Cohen’s *d* = 0.3, at 0.8 power and an alpha of 0.05.

### Participants

Thirty patients with a-MCI were included in this study and were recruited from community announcements or referred by the Old Age Research Group at the Institute of Psychiatry, University of São Paulo.

Before admission in the study, participants underwent clinical and neuropsychological evaluations and a MRI examination. Inclusion criteria were: (1) 60 years of age or older, (2) four or more years of education, (3) right handedness, (4) Portuguese as a native language, (5) normal or corrected vision and hearing, and (6) diagnosis of a-MCI, according to Petersen’s criteria (Petersen et al., 2009). MCI diagnosis was operationalized as follows: (1) presence of subjective memory complaint corroborated by an informant; (2) presence of memory impairment (we considered at least 1.5 standard deviation (SD) below the age norms on one memory test or 1.0 SD below norms on more than one memory test); (3) normal general cognitive function; (4) essentially normal functional of daily living (e) absence of dementia. Exclusion criteria included (1) history of severe neurologic or psychiatry disorder, (2) history of alcohol or drug abuse; (3) and MRI contraindication. Medications were carefully evaluated to exclude those who were using agents that affect cognition; however, antidepressants were allowed as long as the patient had been on a stable dose for at least 6 months without significant mood symptoms. None of the patients were using cholinesterase inhibitors or memantine. Moreover, we also incorporated an established biomarker measurement (i.e., brain volumes) at baseline for investigation of structural inter-group differences, and as a methodological caution, since brain atrophy may affect the BOLD signal in MCI-AD (Johnson et al., 2000; Dickerson et al., 2005; Dickerson and Sperling, 2008).

### Clinical and Neuropsychological Evaluation

The clinical evaluation was conducted by a geriatric psychiatrist and took place in the presence of the patient and an informant, with the exception of four cases that the informant was contacted by telephone. Participants were assessed with the structured clinical interview of the Cambridge Examination for Mental Disorders of Older People (CAMDEX) (Roth et al., 1986; Bottino et al., 1999), Hamilton Anxiety Rating Scale (HAMA) (Hamilton, 1959), Montgomery–Åsberg Depression Rating Scale (MADRS) (Montgomery and Asberg, 1979; Dratcu et al., 1987), Informant Questionnaire on Cognitive Decline in the Elderly (IQCODE) (Jorm and Jacomb, 1989; Perroco et al., 2009) and the Bayer Activities of Daily Living Scale (B-ADL) (Hindmarch et al., 1998; Folquitto et al., 2007). In addition, to assess participant’s handedness, we applied the Annett’s Hand Preference Questionnaire (Annett, 1970).

The neuropsychological assessment was performed by a neuropsychologist and included: Montreal Cognitive Assessment (MoCA) (Nasreddine et al., 2005; Memória et al., 2013), Vocabulary, Matrix Reasoning and Digit Span from the Wechsler Adult Intelligence Scale third edition (WAIS-III) (Wechsler, 1981), Short Cognitive Performance Test (SKT) (Erzigkeit, 2001; Flaks et al., 2009), Stroop Test (Spreen and Strauss, 1998), Phonemic Fluency (letters FAS), Semantic Fluency (animal category) (Benton et al., 1983), Boston Naming Test (BNT) (Kaplan et al., 2001; Miotto et al., 2010), Rey–Osterrieth Complex Figure Test (ROCF) (Rey, 1999; Oliveira et al., 2004), Hopkins Verbal Learning Test Revised (HVLT-R) (Brandt, 1991), Logical Memory and Faces from the Wechsler Memory Scale third edition (WMS-III) (Wechsler, 1997; Nascimento, 2005). An estimate Intellectual Quotient (IQ) measure was calculated from Vocabulary and Matrix Reasoning’s scores (Ringe et al., 2002). Also, measures of metamemory were evaluated through the Multifactorial Memory Questionnaire (MMQ) (Troyer and Rich, 2002; Simon et al., 2016) and self-report mood symptoms through Beck Depression Inventory (BDI) and Beck Anxiety Inventory (BAI) (Beck et al., 1988a,b; Gorenstein et al., 1999).

### Intervention Programs

Two interventions were administered to the participants: MST or EP. Each program was administered individually, with participants attending four 1-h sessions, twice a week, over a 2-week period. All information was presented using a 10-inch tablet. The programs were delivered by neuropsychologists following a step-by-step manual.

#### Mnemonic Strategy Training

Mnemonic strategy training was based on the Ecologically Oriented Neurorehabilitation of Memory (EON-MEM) program (Stringer, 2007). In total, participants were trained in 36 face-name pairs, divided across three training sessions (12 pairs in each session), and in the last session, the participants reviewed all content. Procedures otherwise followed the methods detailed in Hampstead et al. (2008). Briefly, for each face-name pair, patients should follow the “feature-reason-image” process: they were directed to a salient facial feature (visual cue), given a “nickname” linking the facial feature to the name (reason or verbal cue), and then instructed to create a mental image that integrated the feature and reason in a detailed and potentially exaggerated image (e.g., enhancing the salient facial feature so it better stood out). The reasons/verbal cues were phonologically similar or rhymed with the actual name. On each training trial, participants were required to first recall the feature, then the reason, and finally the corresponding name. To reinforce this series of steps, participants were required to recall these steps in order on up to ten training trials per stimulus. A stimulus was removed from circulation if participants recalled this information on three consecutive trials. Once all these initial trials had been completed for each stimulus, all 12 pairs trained during the session were reviewed using the same step-by-step process (same day review). The next training session began with the review of all 12 pairs from the previous session in a different order (delayed review), and then a new set of 12 pairs was trained. The final training session presented all 36 pairs in random order and the participants had to recall the step-by-step process (“feature-reason-image”) across each of three trials.

At the end of each session, patients completed an ecological “generalization step” in order to enhance comprehension and transfer the MST methodology. Participants were required to apply the methodology to real people in their everyday life (e.g., somebody whose name they had trouble recalling or had forgotten at least once). First, they were asked to imagine the face of the person in details, describe it out loud, and then apply the methodology with the therapist – thereby allowing them to create their own associations. In each session, one real-life example was trained and participants were actively encouraged to use the associative methodology in their daily routines.

#### Education Program

In the EP, the therapist presented and discussed relevant topics, such as: healthy aging, memory functioning, aspects that can interfere in memory (e.g., depression, sleep, and stress), risks and protective factors to dementia, such as healthy lifestyle (e.g., diet, physical exercise); and information regarding MCI and Alzheimer’s disease. Review periods were also integrated at the same time points as in the MST group during which the main points were reinforced. Participants also underwent a content review during the last session. Therapists were not allowed to provide any mnemonic strategy to the participants in this group. Importantly, these materials were designed to result in a session length comparable to the MST program.

### Outcome Measures: fMRI and Cognition

#### fMRI Task

Brain activation related to memory encoding was measured using a block design fMRI paradigm. The paradigm was adapted from elsewhere (Hampstead et al., 2011) and comprised alternating 20 s rest, and 24 s active blocks. There were three types of active blocks, including face-name pairs from (1) the trained list, (2) an untrained (i.e., novel) list, and (3) the two control stimuli repeated in alternation. During active blocks, four face-name pairs were shown for 5 s each, with 1 s interstimulus interval. Each block type occurred three times during each of the three functional runs. In total, 72 pairs were displayed once, 36 from each trained and untrained lists; and the control stimuli were seen a total of 36 times. Patients were instructed to press a button as soon as they saw a face-name pair to ensure that they were attending to the stimuli. They were instructed to memorize the name paired with each face. To avoid order bias, block order was different in each run, and run order was counterbalanced across sessions. Participants were familiarized with the task before each session (using the repeated stimuli) with reminder instructions provided prior to each run. The same procedure was performed at pre and post-intervention fMRI sessions, but outside of the scanner at the 1- and 3-month follow-up.

Stimuli presented in the fMRI task were taken from Hampstead et al. (2011) and included a total of 74 faces used in an earlier study (Kirwan and Stark, 2004), transformed to grayscale images, and randomly paired with gender-appropriate names in Portuguese (5–7 letters). As mentioned above, 72 face-name pairs were divided into two lists, which were matched for gender, race, approximate age, and emotional valence (positive, negative, and neutral), and the remaining two faces (one of each gender) served as control stimuli. Stimuli presentation and response recording were performed with E-Prime 2.0 software.

#### Near-Transfer Measures

##### Face-name recognition task

In order to evaluate near-transfer effects (e.g., cognitive operations related to face-name associations), participants completed the Face-Name Recognition Task (FNRT) 30 min after the fMRI paradigm. The task included all 74 stimuli and involved a four-alternative recognition format (Hampstead et al., 2008). These four choices were (1) the target name, (2) a sex-appropriate name from trained list, (3) a sex-appropriate name from the untrained list, and (4) a sex-appropriate novel name. Since the focus of the current work is transfer effects, three measures were selected from the FNRT focusing in the untrained stimuli: (1) accuracy (% correct), (2) confidence ratings for each response using an anchored 4-point scale (1 = not confident at all, 4 = extremely confident), and (3) reaction time (RT). For the follow-up assessments, the procedures were the same, except for the task being presented outside the MRI scan.

##### Strategy use task

The frequency of strategy use was assessed though the Strategy Use Task (SUT). This experimental task required the participants to memorize 12 new face-name pairs presented for 15 s each. The participants were only told to remember the name that matched each face; they were not explicitly instructed to use any strategies. After a 20-min delay, the faces were presented one by one and participants were asked to state the appropriate name (cued recall). Participants then performed a three-choice recognition task (options included the target name, a name from a different pair within this same test, and a novel name). After each response, participants were asked “how” they remembered the name. If their responses include any associative link between face and name, one point was provided. Parallel versions were developed and used for each assessment period in this task.

#### Far-Transfer Measures

Far-transfer effects were assessed through tasks involving episodic memory (not related to face-name material), attention, and metamemory. These constructs were not specifically aimed in the MST, therefore were considered as far transfer measures. The instruments are listed below:

##### Hopkins verbal learning test - revised

The Hopkins Verbal Learning Test - Revised (HVLT-R) is a widely used word list test involving verbal learning and memory, which includes 12 words from three semantic categories. We analyzed the immediate and delayed recall measures. Parallel versions were used in each study assessment (Brandt, 1991).

##### Short cognitive test

The Short Cognitive Test (SKT) is a brief neuropsychological battery involving attention and visual episodic memory. We calculated a composite score of attention based on six attention sub-items, and computed the memory scores for immediate and delayed recalls. In each recall task the participant had to remember 12 color drawings of everyday objects. We computed the number of omissions of each recall. Parallel versions were used in the assessments (Erzigkeit, 2001; Flaks et al., 2009).

##### Multifactorial memory questionnaire

Metamemory was evaluated through the self-report Multifactorial Metamemory Questionnaire (MMQ). The MMQ includes the Contentment scale, assessing participant’s emotions linked to memory performance; Ability scale, to evaluate memory mistakes in everyday life, and Strategy scale, which assess the frequency of using several memory strategies (Troyer and Rich, 2002; Simon et al., 2016).

### Neuroimaging Data Acquisition

#### Magnetic Resonance Imaging

MRI scanning was performed on a 3T Philips Achieva machine MR system. Image acquisition parameters were based on BOLD (Blood Oxygenation Level Dependent) effect: echo planar sequence (EPI GRE) encompassing whole brain (TR = 2000 ms, TE = 30 ms, flip angle 90°, three dummy scans, 40 slices axial oblique AC-PÇ oriented with 3 mm isotropic voxels). Three runs were acquired in the fMRI experiment, each with 199 volumes. A structural 3D T1-weighted scan was acquired immediately after the fMRI acquisition for volumetry brain morphometry (VBM), activation map-coregistration, and normalization procedures described ahead (TR = 7 ms, TE = 3.2 ms, flip angle 8°, 180 slices, 1 mm isotropic voxels). An Axial Fluid-Attenuated Inversion Recovery (FLAIR) scan and susceptibility-weighted (Principles of Echo-Shifting with a Train of Observations – PRESTO) acquisitions – together with T1 acquisition – were used to identify brain alterations. All images followed a quality control protocol and were inspected by a neuroradiologist.

### Data Analysis

#### Functional Neuroimaging

Data processing and statistical analyses were conducted using FMRIB Software Library (FSL) version 6.0 (Smith et al., 2004). Functional volumes were processed by motion correction (MCFLIRT), slice-timing correction using Fourier-space time-series phase-shifting, non-brain removal using BET (Smith, 2002), spatial smoothing (FWHM = 5 mm) and highpass temporal filtering (sigma = 50.0 s), to remove signal drift and low-frequency noise. Time-series statistical analysis was carried out using the general linear model (FILM) with local autocorrelation correction (Woolrich et al., 2004). The functional images were then registered with high resolution structural and standard space images using FLIRT (Jenkinson and Smith, 2001; Jenkinson et al., 2002). For higher-level analysis of multiple sessions (mean of three runs and time effect) we used a fixed effect model, and for multiple subjects (group mean, group comparison and interaction – ANOVA) the FLAME (FMRIB’S Local Analysis of Mixed Effects) was used (Beckmann et al., 2003; Woolrich et al., 2004; Woolrich, 2008). Z statistic images (Gaussianized T/F) were thresholded using clusters determined by *Z*-score > 2.3 and a corrected cluster significance threshold of *p* < 0.05 (Worsley, 2003).

Activation maps were generated for two contrasts. First, to investigate brain activation related to the task, we combined the trained and untrained stimuli from the pre-training scan into a single group of ‘novel’ stimuli [we called ‘novel’ since interventions had yet to take place (Hampstead et al., 2011)]. Activation in response to these stimuli was compared to that for the repeated face-name pairs in the contrast novel > repeated. Second, we used a repeated measure ANOVA, considering factors of time (baseline, 1-week) and group (MST, ED), to identify training effects related to the untrained stimuli, in the contrast: {POST [untrained stimuli > repeated stimuli] > PRE [untrained stimuli > repeated stimuli]} to assess increase of activation after the interventions; and {PRE [untrained stimuli > repeated stimuli] > POST [untrained stimuli > repeated stimuli]} to investigate decrease of activation after the programs. In addition, the percent of BOLD signal change was calculated in the brain areas that emerged in the interactions.

#### Structural Neuroimaging

Brain atrophy, especially affecting medial temporal structures, is commonly observed in a-MCI (Whitwell et al., 2007; Soldan et al., 2015), with evidence that hippocampus and amygdala present the largest annual decline (Fjell et al., 2009). This pattern of brain atrophy may affect the BOLD signal in MCI-AD spectrum (Johnson et al., 2000; Dickerson et al., 2005; Dickerson and Sperling, 2008), thus volumetric differences between the groups could affect data analysis and interpretation of the fMRI results, particularly due to atrophy affecting the hippocampus and amygdala. Therefore, we examined volumetric measurements for all patients with voxel-based morphometry (VBM) using the Statistical Parametric Mapping software (SPM8; Wellcome Department of Imaging Neuroscience, London, United Kingdom) (Ashburner, 2007) (see Supplementary Material for further information). Based on the known patterns of cortical atrophy in a-MCI, we analyzed total brain volumes (gray and white matter) and regional volumes of the hippocampus and amygdala, predicted *a priori* to show brain atrophy based on previous research on MCI (Hampstead et al., 2012a). We then compared (one-way ANOVA) these values to those of an equally sized control sample recruited in the same neuroimaging laboratory in order to demonstrate differences in AD related brain regions.

#### Statistical Analysis

At baseline, comparisons between groups were assessed. It is worth mentioning that between-groups comparisons of brain volumes were treated with the same statistics used for the behavioral data. For categorical variables we used chi-squared tests; and for continuous variables we used the Independent *T*-Test. In order to compare the groups between the different sessions we used repeated measure ANOVA, considering factors of time (baseline, 1-week, 1-month, and 3-months) and group (MST, ED). More specifically, in order to evaluate training-related changes at post-training and maintenance at follow-up, we compared the baseline with each time point of the study. The significance was set at *p* ≤ 0.05. In order to verify the effect-sizes, Cohen’s *d* was calculated. Statistical analyses were performed using the Statistical Package for Social Sciences (SPSS) version 17.

## Results

### Brain Volume, Clinical, and Cognitive Characteristics at Baseline

Table 1 presents the demographical, clinical, and neuropsychological characteristics of the participants, with no significant differences between groups except for the immediate recall of SKT-Memory [*t*(28) = 2.16, *p* = 0.04].

**Table 1:** Demographic, clinical, and neuropsychological characteristics of the sample.

	MST (*n* = 15) *M* (*SD*)	Education *n* = 15 *M* (*SD*)	*p*-Value
**Demographics**			
Age (years)	73.3 (5.9)	71.0 (6.5)	0.31
Education (years)	11.7 (3.6)	12.5 (4.5)	0.57
Sex (females %)	73.3%	80%	1
Ethnicity, Caucasian (%)	53%	60%	1
**Clinical characteristics**			
MADRS	3.6 (3.6)	2.7 (2.9)	0.44
HAMA	3.5 (4.2)	2.4 (2.7)	0.41
IQCODE	3.2 (0.2)	3.0 (0.6)	0.22
B-ADL	1.6 (0.6)	1.7 (0.7)	0.81
Years of memory complaint^∗^	3.7 (2.6)	4.5 (2.6)	0.41
aMCI subtype (MD/SD)	10/5	12/3	0.68
**Neuropsychological performance**			
MoCA	24.3 (2.2)	23.9 (2.7)	0.71
Estimated IQ	97.9 (7.7)	97.3 (10.7)	0.86
Digit span forward (WAIS-III)	7.4 (1.6)	8.0 (2.0)	0.37
Digit span backward (WAIS-III)	4.2 (1.3)	4.8 (1.5)	0.25
SKT-Attention score	1.6 (2.0)	2.3 (2.2)	0.36
SKT-Memory immediate recall	6.7 (0.9)	5.7 (1.5)	0.04
SKT-Memory delayed recall	6.20 (1.7)	6.2 (2.0)	1
Stroop (time on third plate)	34.7 (9.6)	41.1 (15.7)	0.23
HVLT-R immediate recall	20.8 (2.9)	20.5 (3.8)	0.83
HVLT-R delayed recall	3.5 (2.8)	2.7 (2.4)	0.41
Faces immediate recall (WMS-III)	33.2 (4.8)	34.2 (3.7)	0.53
Faces delayed recall (WMS-III)	31.2 (4.7)	33.1 (3.2)	0.20
LM immediate recall (WMS-III)	19.5 (5.7)	22.3 (5.8)	0.20
LM delayed recall (WMS-III)	16.9 (5.3)	15.8 (8.0)	0.67
ROCF	29.8 (3.4)	27.1 (5.0)	0.10
COWAT (FAS)	35.2 (10.0)	33.5 (11.7)	0.67
Semantic fluency (animal)	15.8 (3.6)	13.6 (3.3)	0.10
Boston Naming Test	54.9 (4.8)	50.9 (7.2)	0.09

*B-ADL, Bayer Activities of Daily Living Scale; COWAT, Controlled Oral Word Association Test; HAMA, Hamilton Anxiety Rating Scale; HVLT-R, Hopkins Verbal Learning Test Revised; IQ, Intelligence Quotient; IQCODE, Informant Questionnaire on Cognitive Decline in the Elderly; LM, Logical Memory; M, mean; MADRS, Montgomery–Åsberg Depression Rating Scale; MD/SD, Multiple/Single Domain; MoCA, Montreal Cognitive Assessment; MST, memory strategy training; ROCF, Rey–Osterrieth Complex Figure Test; SKT, Short Cognitive Performance Test; SD, standard deviation; WAIS, Wechsler Adult Intelligence; WMS, Wechsler Memory Scale; ^∗^self-report.*

Regarding the brain volume data, the one-way ANOVA revealed that the MCI sample presented significant gray matter volume decrements relative to control sample in regions typically affected by Alzheimer’s pathology, including the bilateral hippocampus [*F*(1,58) = 14.2, *p* < 0.001], and amygdala [*F*(1,58) = 68.9, *p* < 0.001] (Table 2). Importantly, there were no differences in these volumes, and total brain volume, between the a-MCI treatment groups (Table 3).

**Table 2:** Volumetric measures in the MCI study sample and in a control sample^∗^.

	MCI (*n* = 30) *M* (*SD*)	Control (*n* = 30) *M* (*SD*)	*p*-Value
Age	72.1 (6.2)	72.4 (5.9)	0.84
Education	12.1 (4.0)	11.3 (5.6)	0.54
Sex (F/M)	24/6	24/6	1
Amygdala (R and L)	0.00178 (0.00007)	0.00201 (0.00012)	<0.001
Hippocampus (R and L)	0.00610 (0.00040)	0.00651 (0.00042)	<0.001

*^∗^The two samples were collected, processed and analyzed in the same neuroimaging laboratory. L, left; M, mean; SD, standard deviation; R, right. Volumetric measures represented in ml.*

**Table 3:** MRI Volumetry (ml) for each intervention group.

	MST (*n* = 15) *M* (*SD*)	ED (*n* = 15) *M* (*SD*)	*p*-Value
Total volume (gray + white matter)	1026.861 (90.0)	1053.996 (98.7)	0.43
Amygdala (R and L)	0.00179 (0.00008)	0.00177 (0.00007)	0.53
Hippocampus (R and L)	0.00614 (0.00044)	0.00606 (0.00035)	0.60

*ED, Education; L, left; M, mean; MST, memory strategy training; SD, standard deviation; R, right.*

### Trained-Relates Changes

#### Near-Transfer Effects

##### Untrained stimuli (FNRT)

**FIGURE 2:**
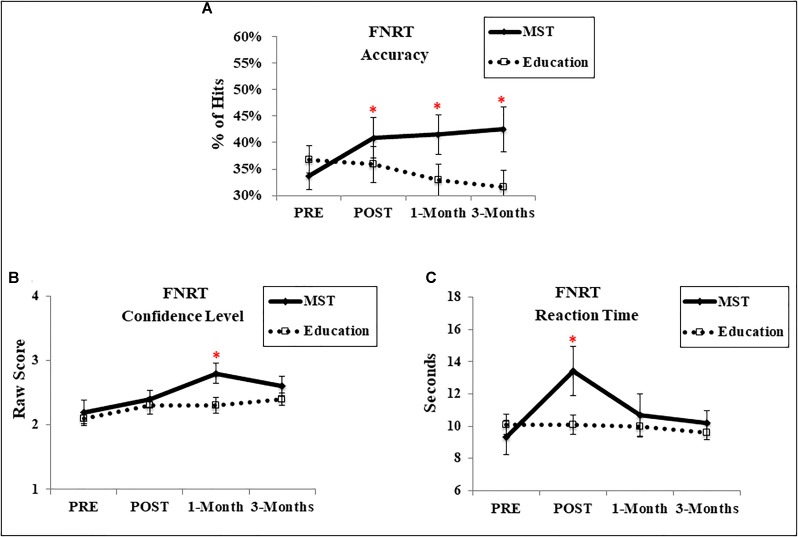
Performance in the FNRT. **(A)** Accuracy; **(B)** confidence level of response; **(C)** reaction time. ^∗^ Represents *p* < 0.05 indicating a time-by-group interaction. Error bars represent standard error mean. FNRT, Face-name recognition task; MST, memory strategy training.

Regarding accuracy, after 1-week there was no main effect of time [*F*(1,28) = 2.41, *p* = 0.13], or group [*F*(1,28) = 0.05, *p* = 0.81]; however, we observed a significant time-by-group interaction [*F*(1,28) = 4.04, *p* = 0.05; *d* = 0.35) (Figure 2A). This interaction was driven by accuracy improvement for the MST [*t*(14) = −2.27, *p* = 0.03] but not the ED group [*t*(14) = 0.36, *p* = 0.71]. This pattern was more evident at follow-up evaluations, when we observed significant time-by-group interactions (1-month: [*F*(1,28) = 6.38, *p* = 0.01]; and 3-months: [*F*(1,27) = 8.38, *p* = 0.007]), and greater effect-sizes (1-month: *d* = 0.64; 3-month: *d* = 0.75), suggesting that near transfer-effects persisted at follow-up. Analysis of RT revealed a main effect of time [*F*(1,28) = 8.83, *p* = 0.006] and time-by-group interaction, indicating a slower performance in the MST group at post-intervention [*F*(1,28) = 8.41, *p* = 0.007; *d* = 0.79] (Figure 2B). However, no interactions were observed at 1-month [*F*(1,28) = 3.06, *p* = 0.09; *d* = 0.17] or 3-months follow-up [*F*(1,27) = 1.38, *p* = 0.25; *d* = 0.27].

For confidence (Figure 2C), there was a main effect of time at 1-week [*F*(1,28) = 6.87, *p* = 0.01] but no effect of group [*F*(1,28) = 0.27, *p* = 0.60] or time-by-group interaction [*F*(1,28) = 0.21, *p* = 0.88; *d* = 0.21]. Instead, an interaction emerged at 1-month [*F*(1,28) = 4.34, *p* = 0.04; *d* = 0.89], indicating greater confidence in the MST group; which dissipated at 3-months follow-up [*F*(1,27) = 1.26, *p* = 0.27; *d* = 0.49].

##### Frequency of strategy use (SUT)

**FIGURE 3:**
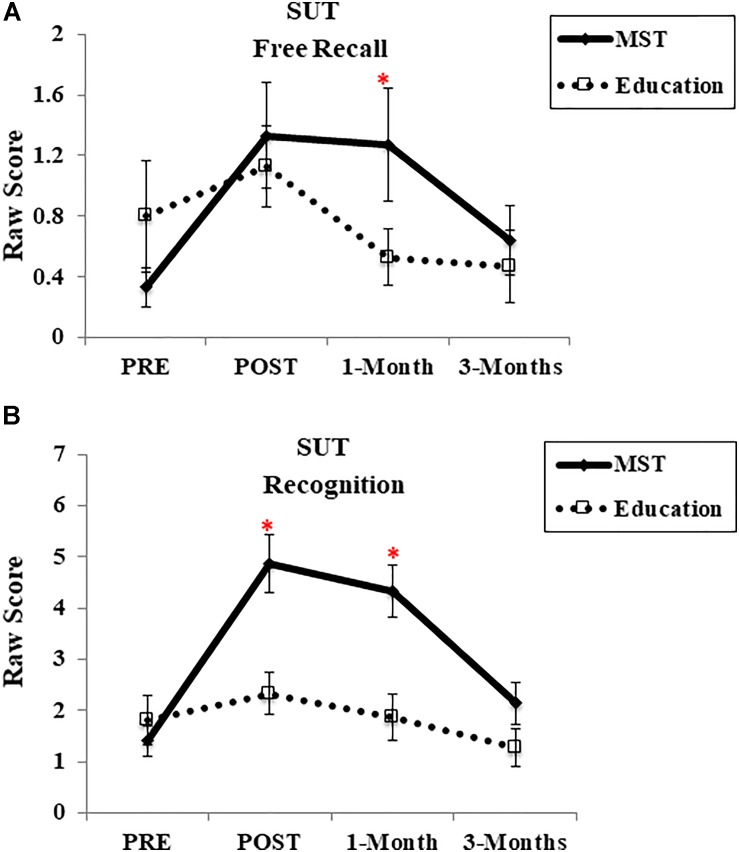
Frequency of strategy use. **(A)** SUT – Free recall; **(B)** SUT – Recognition Task. MST, mnemonic strategy training; SUT, strategy use task. ^∗^Represents; *p* < 0.05 indicating time-group interactions. Error bars represent standard error mean.

The frequency of strategy use for new face-name pairs was measured in the context of free recall and recognition. One-week after the programs, there was a time-by-group interaction for the recognition task [*F*(1,28) = 18.53, *p* < 0.001; *d* = 1.33] (Figure 3A), but not for the cued recall [*F*(1,28) = 2.63, *p* = 0.11; *d* = 0.17] (Figure 3B). The interaction for the recognition task was driven by the fact that only the MST group showed significant improvement after the intervention [*t*(14) = −5.85, *p* < 0.001], whereas the control group did not [*t*(14) = −1.58, *p* = 0.13]. Moreover, at 1-month follow-up, we observed an interaction for both free recall [*F*(1,28) = 7.58, *p* = 0.01; *d* = 0.67] and recognition [*F*(1,28) = 11.51, *p* = 0.002; *d* = 1.33], however, it dissipated after 3-months [cued recall: *F*(1,27) = 2.19, *p* = 0.15; *d* = 0.20; recognition: *F*(1,17) = 3.46, *p* = 0.07; *d* = 0.58].

#### Far-Transfer Effects

##### Neuropsychological tests (HVLT-R and SKT)

**FIGURE 4:**
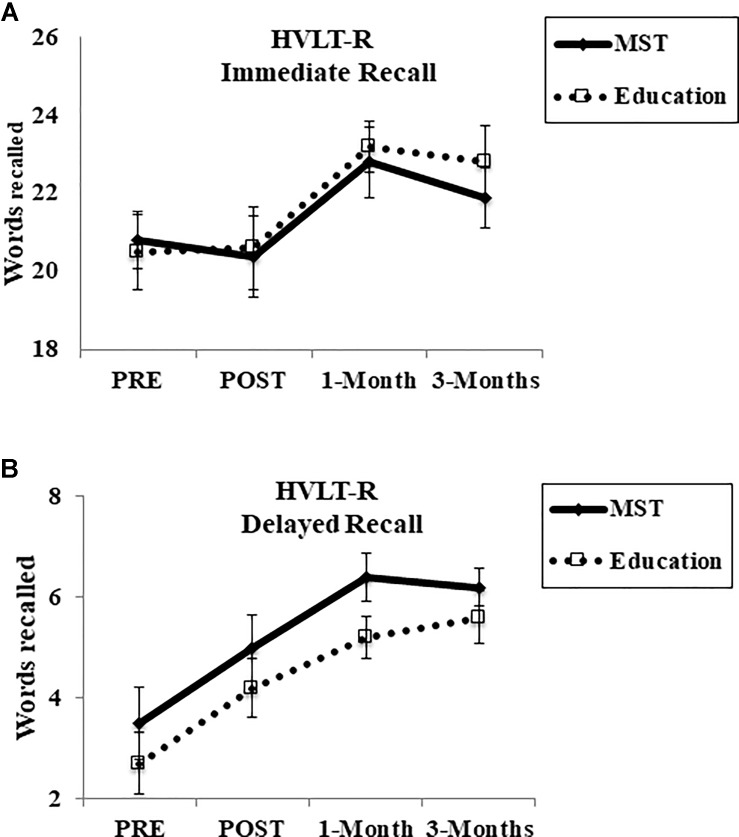
Cognitive performance in HVLT-R. **(A)** Immediate recall; **(B)** delayed recall. HVLT-R, Hopkins Verbal Learning Test-Revised; MST, mnemonic strategy training. Error bars represent standard error mean.

Regarding HVLT-R immediate recall, at 1-week there was no main effect of time [*F*(1,28) = 0.26, *p* = 0.61], group [*F*(1,28) = 0.18, *p* = 0.66], or interaction [*F*(1,28) = 0.10, *p* = 0.74; *d* = 0.18] at any time point (1-month: [*F*(1,28) = 0.45, *p* = 0.50; *d* = 0.13]; 3-month: [*F*(1,27) = 0.52, *p* = 0.47; *d* = 0.29]) (Figure 4A). For the HVLT-R delayed recall, at post-intervention we observed a main effect of time [*F*(1,28) = 5.70, *p* = 0.02], but no effect of group [*F*(1,28) = 1.31, *p* = 0.26] or time-by-group interaction [*F*(1,28) < 0.03, *p* = 0.95; *d* = 0.31] at any follow-up assessment [1-month: *F*(1,28) = 0.23, *p* = 0.63; *d* = 0.69; 3-months: *F*(1,27) = 0.01, *p* = 0.91; *d* = 0.40] (Figure 4B).

**FIGURE 5:**
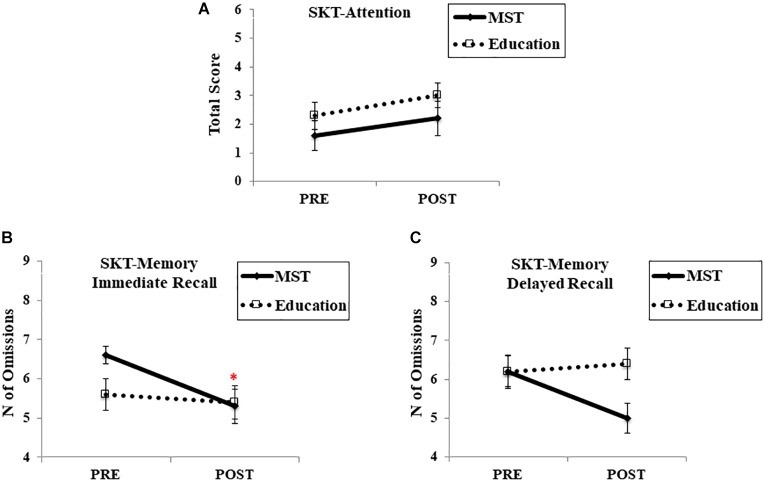
Cognitive performance in SKT. **(A)** SKT-Attention score; **(B)** SKT-Memory immediate recall; **(C)** SKT-Memory delayed recall. MST, mnemonic strategy training; SKT, Short Cognitive Test. ^∗^ Represents *p* < 0.05 indicating a time-by-group interaction. Error bars represent standard error mean.

For the SKT-Attention score, there was no effect of time [*F*(1,28) = 2.14, *p* = 0.15], group [*F*(1,28) = 0.23, *p* = 0.63], or group-by-time interaction [*F*(1,28) > 1; *p* = 0.93; *d* = 0.46] (Figure 5A). Although we did not observe a main effect of group in the SKT-Memory immediate recall [*F*(1,28) > 1; *p* = 0.93], there was a main effect of time [*F*(1,28) = 7.74; *p* = 0.01] and group-by-time interaction [*F*(1,28) = 4.23; *p* = 0.04; *d* = 0.08], indicating a slightly better performance (fewer omissions) in the MST group at post-intervention (Figure 5B). Moreover, in the SKT-Memory delayed recall, there was no effect of time [*F*(1,28) = 1.82; *p* = 0.18] or group [*F*(1,28) = 1.96; *p* = 0.17] though there was a trend for the interaction [*F*(1,28) = 3.58; *p* = 0.06; *d* = 0.9] (Figure 5C). It is worth mentioning that SKT was not re-assessed at follow-up due to a limited number of test versions.

##### Metamemory questionnaire (MMQ)

**FIGURE 6:**
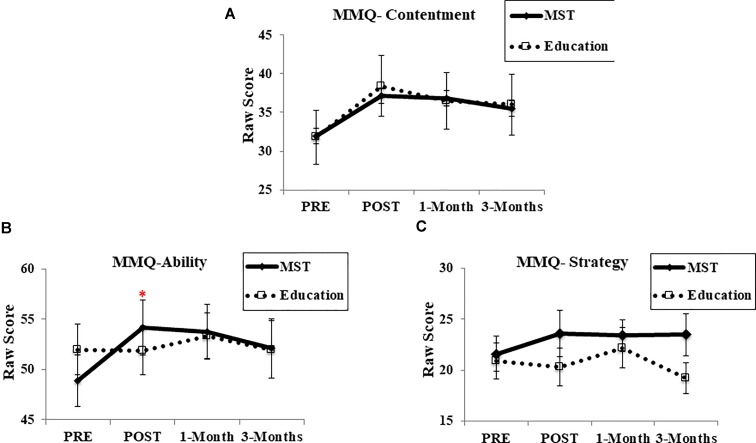
Performance in metamemory questionnaire. **(A)** MMQ-Contentment scale; **(B)** MMQ-Ability scale; **(C)** MMQ-Strategy scale. MMQ, Multifactorial Memory Questionnaire; MST, mnemonic strategy training. ^∗^ Represents *p* < 0.05 indicating a time-by-group interaction. Error bars represent standard error mean.

In the MMQ-Feelings post-training subscale, an effect of time [*F*(1,28) = 12.43, *p* > 0.001] suggested that both groups reported greater contentment about their own memory after the programs (Figure 6A). However, there was no main effect of group [*F*(1,28) = 0.01, *p* = 0.91] or interaction [*F*(1,28) = 0.15, *p* = 0.69; *d* = 0.08], including at the follow-up assessments [1-month: *F*(1,28) = 0.37, *p* = 0.54; *d* = 0.02; 3-months: *F*(1,27) = 1.46, *p* = 0.23; *d* = 0.03]. For MMQ-Ability, although there was no main effect of group at post-training [*F*(1,28) = 0.16, *p* = 0.90]; we observed an effect of time [*F*(1,28) = 6.47, *p* = 0.01] and time-by-group interaction [*F*(1,28) = 7.16, *p* = 0.01; *d* = 0.23], indicating that only the MST group reported greater memory ability in everyday life after the programs; however, this did not persist at follow-up evaluations [1-month: *F*(1,28) = 0.65, *p* = 0.42; *d* = 0.05; 3-months: *F*(1,27) = 1.09, *p* = 0.30; *d* = 0.05] (Figure 6B). In the MMQ-Strategy, there was no main effect of time [*F*(1,28) = 0.28, *p* = 0.60], group [*F*(1,28) = 0.68, *p* = 0.41], nor time-by-group interaction [*F*(1,28) = 0.96, *p* = 0.33; *d* = 0.41] either at post-treatment or at follow-up [1-month: *F*(1,28) = 0.75, *p* = 0.39; *d* = 0.17; 3-months: *F*(1,27) = 0.18, *p* = 0.67; *d* = 0.62] (Figure 6C).

### Neuroimaging Data

#### Brain Activation Related to the fMRI Task

**FIGURE 7:**
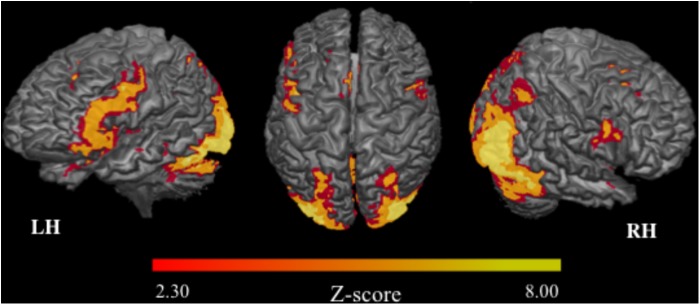
Brain activation related to the fMRI task (prior to intervention). 3D maps: mean of activation in the contrast Novel stimuli > Repeated stimuli. LH, left hemisphere; RH, right hemisphere; *n* = 30; *Z*-score: >2.30; *p* < 0.05.

At pre-training, increase of activation in several brain foci was evident when subjects were encoding novel relative to repeated face-name stimuli, including widespread bilateral regions within the occipital and parietal cortices, the cerebellar vermis, intraparietal sulcus and posterior portions of the fusiform gyrus (consistent with the fusiform face area, e.g., Solomon-Harris et al., 2016). Regarding the frontal cortex, we observed the activation in bilateral regions such as inferior frontal and precentral gyri; in left areas, such as middle frontal, orbital gyrus, and anterior insula; and in medial structures, including the anterior cingulate gyrus and medial frontal gyrus. Finally, increased activity was also found in the bilateral hippocampal formation, amygdala and putamen (Figure 7 and Table 4A).

**Table 4:** Statistical information of significant clusters highlighted prior to intervention.

Contrast	Region^1^ (Brodmann Area)	Side	MNI coordinates			Cluster size	*Z*	Cluster *p*-value^2^
			*x*	*y*	*z*			
**(A)** Novel > repeated	Widespread occipital	L	−40	−58	−24	28677	7.1	<0.0001
	Inferior frontal gyrus (6,44)	L	−44	04	34	3263	5.13	<0.0001
		R	40	04	24	631	3.87	0.0025
	Anterior cingulate (32)	R/L	0	22	36	1570	4.67	<0.0001
**(B)** Novel > repeated	Cuneus (17)	L	−2	−94	6	553	3.52	0.0059
MST > EP								

*^1^Peak of cluster activation; ^2^values corrected for multiple comparisons; MST, memory strategy training; EP, education program.*

In order to investigate group differences in brain activation related to encoding of stimuli, the groups were compared in the same contrast (novel > repeated stimuli). We found that the MST group showed greater activation only in the right cuneus, part of the primary visual cortex (Table 4B).

#### Brain Activation Related to the Training

**FIGURE 8:**
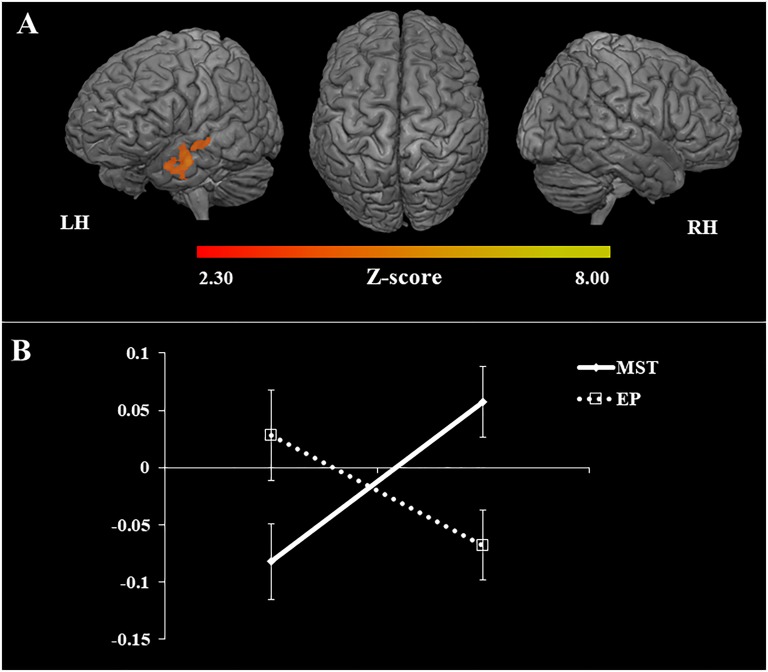
Changes in task-related brain activation and % of BOLD signal. **(A)** 3D maps: mean of activation revealed by the ANOVAs. Increase of brain activation (fMRI task) related to the untrained stimuli, in the contrast Post [Untrained stimuli > Repeated stimuli] > Pre [Untrained stimuli > Repeated stimuli]. LH, left hemisphere; RH, right hemisphere; *n* = 30; *Z*-score: >2.30; *p* < 0.05; **(B)** graph: % of BOLD signal at pre and post intervention in the superior temporal sulcus (region of peak of activation), EP, education program; MST, mnemonic strategy training.

Brain activation related to the training effect was investigated through the repeated measure ANOVA. Our findings revealed that after the interventions the MST group showed a greater increase of activation than EP in multiple regions of the left anterior temporal lobe. The peak of activation occurred in the left superior temporal sulcus (STS) (Brodmann area 38) with signal change extending to the middle temporal gyrus (MTG) and superior temporal gyrus (STG) (Table 5 and Figure 8A). In addition, BOLD signal in the STS increased in the MST group, but declined in the EP group (Figure 8B).

**Table 5:** Statistical information of significant clusters highlighted in the ANOVAs.

Contrast	Region^1^ (Brodmann Area)	Side	MNI coordinates			Cluster size	*Z*	Cluster *p*-value^2^
			*x*	*y*	*z*			
Post > pre	Superior temporal sulcus (38)	L	−52	2	−22	538	3.93	0.0068
MST > EP								
(untrained > repeated)								

*^1^Peak of cluster activation; ^2^values corrected for multiple comparisons; MST, memory strategy training; EP, education program.*

## Discussion

In this randomized, controlled, single-blind study we report the efficacy of MST focused on face-name stimuli in patients with a-MCI. Our experimental design allowed us to observe that learning mnemonic strategies were more beneficial to memory performance than learning non-specific contents in an active control group (EP). We found that only after MST there was a significant improvement in memory performance for untrained face-name pairs, as well as increase of strategy use when recalling new face-name pairs, which persisted at follow-up. Interestingly, only participants in the MST group reported an increase of self-rated memory ability at post-intervention. Despite such findings, we did not observe consistent changes in neuropsychological measures of attention or episodic memory (SKT and HVLT-R), except for the SKT immediate recall.

Our neuroimaging data corroborate the behavioral findings, showing that only after MST there was an increase of task-related brain activation in brain regions known to be implicated in several of the cognitive processes trained. It is relevant to highlight that our findings were not confounded by between-group differences in terms of brain atrophy despite the presence of significantly reduced volume in key MTL regions. Such findings underscore our conclusion that MST may be efficacious in a-MCI due to AD.

### Near-Transfer Effects

Our findings are highly suggestive of near-transfer effect after cognitive training, which is in line with several studies with healthy older adults and MCI patients (for a recent review, see Mewborn et al., 2017; and a recent work Belleville et al., 2018). We found that 1-week after the programs, the MST group presented a greater improvement in accuracy for untrained stimuli, which were maintained at follow-up evaluations. Curiously, the MST group’s confidence increased as they were further removed from training. This likely reflects (1) an initial non-specific benefit of EP that rapidly dissipated at follow up and (2) providing patients with “tools” (i.e., MST) to overcome an area of weakness has long-term benefit. The observed differences in accuracy (MST better than EP) and reaction time (MST slower than EP) further support this idea. While slowed reaction time may initially seem counterintuitive, it fits with the inherently slower and more effortful nature of using mnemonic strategies (see Hampstead et al., 2014 for discussion).

Moreover, the data regarding the frequency of strategy use (SUT) support the hypothesis that MST group transferred the strategy use to a new context, and maintained it after 1-month. However, the frequency of strategy use decreased to baseline level after 3-months, which suggest that booster sections are desirable to keep mnemonic strategies. It is relevant to note that objective strategy use outcomes after MST are still scarce in the literature, and it is a critical aspect to be further investigated, especially because people with MCI shows impaired strategy use (Ramakers et al., 2010).

Beside our behavioral data, the neuroimaging findings provided robust evidence of near-transfer effect after MST, since only the training group showed increase of brain activation in a left lateral temporal area when encoding untrained face-name pairs, which is consistent with previous studies (Belleville and Bherer, 2012). This area included the left STS, STG, and MTG, traditionally implicated in auditory and language processing, but critically involved in social cognition and face processing (Hein and Knight, 2008; Deen et al., 2015). The STS has been linked to theory of mind (Deen et al., 2015), mirror neuron system (Rizzolatti and Craighero, 2004; Molenberghs et al., 2010), process of social cues (Perrett et al., 1992), face processing (Pitcher et al., 2011), face recognition (Sliwinska and Pitcher, 2018) and perception of physical features such as attractiveness. Similarly, the MTG is implicated in observation of facial expressions (Sato et al., 2012), and STG in the perception of emotions and changeable characteristics of a face (Bigler et al., 2007). Interestingly, these aspects are consistent with the visual cues (i.e., “Feature”) that participants learned in the face-name association training. Part of the cues provided relied on physical characteristics, but others in the emotion expression of the face, which may also integrate cognitive processes related to social cognition and empathy. It is likely that our generalization step may have contributed to the near-transfer effect observed, since we reinforced the associative strategy with real and meaningful examples; nevertheless further investigations are necessary to test this hypothesis. In addition, the left STS serves for multiple functions supporting semantic memory and associative thinking (Liebenthal et al., 2014), and the MTG has been linked to semantic memory retrieval (Tranel et al., 1997; Chao et al., 1999), and demanding semantic decisions (Whitney et al., 2011). Thus, it is possible that MST enhanced new learning/memory via additional semantic processing; an approach that could leverage the relatively better preserved semantic network in those with MCI (Murphy et al., 2008). It is worth mentioning that the pattern of activation related to the task (i.e., prior to training) showed activation in a widespread bilateral network, including target areas relevant to episodic memory (i.e., hippocampal formation and amygdala) (Whitney et al., 2011; Soldan et al., 2015), and face processing (i.e., fusiform face area) (Solomon-Harris et al., 2016), indicating that our fMRI task was successful. The brain changes related to transfer effects are coherent with the intervention format and involved the activation of new alternative brain areas. Our finding agrees with models of brain compensation in aging and suggests that maintaining optimal memory functioning relies on both increased activation of specialized areas and recruitment of new alternative brain networks (Cabeza, 2002; Reuter-Lorenz and Cappell, 2008).

### Far-Transfer Effects

It is well established that far-transfer effects are more challenging to be demonstrated than near-transfer effects (Simons et al., 2016; Mewborn et al., 2017). In our study we observed limited evidence of transfer effects on neuropsychological tests of attention and memory (not involving face-name associations). Specifically, there were no changes on measures of SKT-attention, contrary to a previous study (Brum et al., 2009), which can be explained by the fact that the patients were relatively intact at baseline and we did not have any explicit attentional component in the training program. Likewise, there were no changes on the HVLT-R, though this is likely understandable since the MST approach did not teach generalization to word lists (note though that Belleville et al., 2011 have shown MST-based improvement on word lists using a semantic clustering approach that is appropriate for word lists). As discussed earlier (Hampstead et al., 2014), the format of some memory tests may be incompatible with the slower and more demanding nature of MST (i.e., it may be difficult for patients to apply strategies when faced with a stimulus every second). We did, however, see some tenuous evidence of improvement on the SKT during both immediate (*p* = 0.04) and delayed memory (*p* = 0.06) that was specific to the MST group. It is possible that participants were able to use the “FRI” approach to remember the objects used as stimuli in the SKT, though this awaits empirical confirmation. In hindsight, it is possible our selected outcome measures were not optimal for assessing far-transfer so we encourage future studies to thoroughly consider the correspondence between the training approach and cognitive processes being assessed by selected outcome measures. As we previously stated, it is probably unreasonable to expect a single cognitively oriented treatment to effectively address all cognitive domains (or even multiple aspects of a given domain; Hampstead et al., 2014) since, by comparison, one would not consider a medication ineffective if it reduced low density lipoproteins (LDLs) but not very LDLs or failed to increase high density lipoproteins (HDLs). We maintain that transfer effects will be most likely when the training condition approximates deficits encountered in everyday life; an approach that would readily facilitate the clinical translation of research.

Regarding metamemory, both groups reported greater satisfaction with their own memory after intervention, which is both consistent (Troyer et al., 2008; Kinsella et al., 2009) and inconsistent with other studies (Jean et al., 2010; Bahar-Fuchs et al., 2017). One explanation to this finding is that cognitive training and education may have an effect on psychological wellbeing (e.g., providing a sense of control) and arise from interacting with study team members and participating in an intervention program. Our finding that only the MST group reported improved self-rated memory ability again suggests a training specific, near-transfer effect that aligns with prior research (Rapp et al., 2002; Belleville et al., 2006), though this is certainly not a universal finding (Troyer et al., 2008; Kinsella et al., 2009; Jean et al., 2010; Bahar-Fuchs et al., 2017). It is possible that metamemory gains facilitate the transfer to everyday life, since metamemory has been associated with everyday memory performance (McDougall and Kang, 2003). However, this effect dissipated after 1-month, which may suggest that four sessions of MST have an immediate effect on metamemory, but are not enough to maintain the subjective sense of memory-ability improvement over the time. Thus, booster sessions would clearly be needed prior to 1 month to maintain such effects. Although, we found improvement in the frequency of the strategy use on the SUT, we did not observe changes on the self-rated strategy use, which is at odds with previous reports (Rapp et al., 2002; Troyer et al., 2008; Kinsella et al., 2009; Jean et al., 2010; Bahar-Fuchs et al., 2017). In retrospect, we suspect that the focused nature of our intervention explains this discrepancy since these other studies a range of strategies that would be more likely captured by the MMQ-Strategy scale.

Although we observed limited far-transfer effects in our study, as hypothesized, it is especially encouraging that our brief four-session training led to significant improvements in objective and subjective memory measures, as well as robust increase of task-related brain activation. Our findings are in line with previous reports on the underlying mechanism of near-transfer effect of MST (Belleville et al., 2011; Hampstead et al., 2011, 2012b). We believe that the success of our program is related to the focused and intense nature of the training since we provided hundreds of trials in order to reinforce the MST approach. Moreover, we believe the ‘generalization step’ helped participants consolidate the laboratory based training since participants learned to apply the steps using real-life examples.

As with any study, there are some limitations that warrant discussion. First, the findings reported herein are based on a relatively modest sample size, which limits the power of analysis, and calls for replication with larger groups. We are, however, encouraged by the general replication of results across studies. Second, although we showed that our sample presented significant more brain atrophy than a control sample, we classified a-MCI according to clinical criteria, and not based on direct amyloid- and tau-based biomarker data (e.g., PET or CSF). It is critical that future studies include MCI population characterized also based on such AD-related biomarker data in order to better clarify the efficacy of MST in MCI individuals with the same underlying pathological process (i.e., MCI due to AD). Third, due to the sample size, our study was not able to address moderating factors that may predict intervention response, which is critical for identifying those MCI individuals most likely to benefit from cognitive training (Belleville et al., 2018). Last, although others have used education (Vidovich et al., 2015) as an active control condition, we were not able to control for face-name exposure between the groups.

## Conclusion

In conclusion, this randomized controlled study indicates that patients with a-MCI can learn to apply new strategies, which engage task-relevant brain regions, thereby joining other recent works in this area (Belleville et al., 2011; Hampstead et al., 2011, 2012a,b; Rosen et al., 2011; Balardin et al., 2015). Future studies should build on these encouraging findings to investigate and facilitate transfer effects to meaningful everyday life activities.

## Author Contributions

SS, CB, BH, RÁ, and EA: conceptualization. SS, MN, LF, and FP: data collection. FD, LT, and MN: data processing. SS, MN, MM, FD, and CM: data analysis. SS and MN: visualization. SS, CB, and GB: resources. CB, BH, GB, and EA: supervision. SS: wrote the original draft. SS, CB, BH, GB, EA, MN, FD, LF, MM, RÁ, FP, SB, CM, and LT: reviewed and edited the draft.

## Conflict of Interest Statement

The authors declare that the research was conducted in the absence of any commercial or financial relationships that could be construed as a potential conflict of interest.
