# Supplementary material for: Cognitive and Brain Activity Changes After Mnemonic Strategy Training in Amnestic Mild Cognitive Impairment: Evidence From a Randomized Controlled Trial
Source: Front Aging Neurosci. 2018 Nov 13;10:342. doi: 10.3389/fnagi.2018.00342 (PMC6243115; doi:10.3389/fnagi.2018.00342)
Supplement: Supplementary file 1 [file Data_Sheet_1.DOC]

**Supplementary Material**

**Volumetry data**

**Methods**

*Voxel-based morphometry (*VBM) processing was performed using the Statistical Parametric Mapping (SPM), Version 8 (Wellcome Trust Centre of Neuroimaging, London, United Kingdom), implemented in MATLAB R2012a (MathWorks, Sherborn, Massachusetts). First, all anatomical images were reoriented; the mm coordinate of the anterior commissure matched the x y z origin (0,0,0), and the orientation approximated Montreal Neurological Institute (MNI) space. The images were then segmented into GM, WM and cerebrospinal fluid (CSF) partitions using the unified segmentation procedure (Ashburner et al., 2005). The Diffeomorphic Anatomical Registration Through Exponentiated Lie Algebra (DARTEL) algorithm was then used to spatially normalize the segmented images, by registering individual structural images to an asymmetric T1-weighted template derived from participants' structural images (Ashburner, 2007). These fully normalized images were resliced through trilinear interpolation to a final voxel size of 1.5x1.5x1.5 mm³. An additional “modulation” step consisted of multiplying each spatially normalized GM and WM images by their relative volumes before and after normalization.

**Analysis**

We used the SPSS package to investigate the possibility that the two groups would have presented, at baseline, significant volumetric differences (by t-tests) in the following indices: total brain volume (gray and white matter), and hippocampal and amygdala gray matter volumes (normalized to total brain volumes). In each subject, the measure of total gray matter and white matter in the brain were obtained by get totals script (http://www.cs.ucl.ac.uk/staff/g.ridgway/vbm/get_totals.m) implemented for SPM8 on the native space gray and white segmentations for each subject. Measures of gray matter volumes in the hippocampus and amygdala were obtained using the spatially normalized ROI masks that are available within the Anatomical Automatic Labeling SPM toolbox (<http://www.gin.cnrs.fr/AAL>). The gray matter volumes in the hippocampus and amygdala were extracted from the spatially normalized images of each subject, using a script from the SPM mailing list (https://www.jiscmail.ac.uk/cgi-bin/webadmin?A2=spm;3d3a3add.0809).
